# Periprocedural Skincare for Nonenergy and Nonablative Energy‐Based Aesthetic Procedures in Patients With Skin of Color

**DOI:** 10.1111/jocd.16712

**Published:** 2025-01-19

**Authors:** Andrew F. Alexis, Anneke Andriessen, Renée A. Beach, Valeria Barreto Campos, Lisa R. Ginn, Rodrigo Gutiérrez Bravo, Levashni Naidoo, Monica Li

**Affiliations:** ^1^ Clinical Dermatology at Weill Cornell Medicine New York USA; ^2^ Radboud UMC Nijmegen Andriessen Consultants Malden The Netherlands; ^3^ DermAtelier, Faculty of Medicine University of Toronto Ontario Canada; ^4^ University of Jundiai São Paulo Brazil; ^5^ eSkin@LRG Bethesda Maryland USA; ^6^ Private Practice Mexico City Mexico; ^7^ Private Practice, The Dermatology Room Johannesburg South Africa; ^8^ Department of Dermatology and Skin Science University of British Columbia Vancouver Vancouver British Columbia Canada

**Keywords:** Periprocedural, Skincare, SOC

## Abstract

**Background:**

Anti‐aging facial procedures with nonenergy and nonablative energy devices are increasingly popular among patients with skin of color (SOC). Algorithms have addressed the measures to reduce the side effects related to aesthetic procedures, but few focus on SOC patients and periprocedural integrating skincare.

**Methods:**

Eight dermatologists from Brazil, Canada, South Africa, Mexico, and the USA participated in a meeting and an online follow‐up to develop an algorithm for periprocedural skincare for nonenergy and nonablative energy‐based facial aesthetic procedures in patients with SOC. A Delphi method was used to develop this algorithm and integrate information from the literature with panels' clinical experience and opinion, resulting in the current algorithm.

**Results:**

The algorithm has five sections, starting with a medical history and skin examination, followed by pretreatment measures beginning 2–4 weeks before the procedure, then measures on the day of the procedure, aftercare 1–7 days after the procedure, and follow‐up care 1–4 weeks after the procedure and ongoing.

**Conclusions:**

This algorithm provides guidelines for treatment optimization of non‐energy, non‐ablative energy‐based devices for SOC patients. It also provides physicians with skincare recommendations pre‐, peri‐, and post‐aesthetic procedures.

**Abbreviations**
AzA: azelaic acid
HA: hyaluronic acid
HOCl: hypochlorous acid
KA: kojic acid
PA: pigment alterations
SC: stratum corneum
SOC: skin of color
SPF: sun protection factor
SPT: fitzpatrick skin phototype
TXA: tranexamic acid

## 1 Introduction

Demographic shifts and advances in minimally invasive and nonablative technologies have opened aesthetic procedures to a larger patient population [1]. Over the past two decades, the patient population undergoing cosmetic procedures has become increasingly diverse and includes a growing proportion of patients that have skin of color (SOC) [1, 2].

Fitzpatrick skin phototype (SPT) was developed to assess the photosensitity to ultraviolet light and initially included SPT I to IV, whereas phototypes SPT V and VI were added later to include individuals with brown or black skin color [3]. Healthcare providers often use SPT as a proxy for skin tone and predictor of responses to laser and other procedures, which was not the original intent. Notwithstanding the limitations of Fitzpatrick's SPT (including its subjectivity and potential lack of correlation with treatment responses) and numerous proposals for alternative skin classification systems, SPT continues to be the most widely used system by dermatologists globally. The term SOC is used to describe the skin characteristics of the diverse range of populations who self‐identify as non‐white; patients with SOC generally fall within the SPT range of III‐VI [4]. The majority of published cosmetic procedure data in SOC involves patients of East Asian descent with SPT III and IV. By contrast, there is minimal data involving populations with SPT V and VI [5, 6, 7, 8, 9].

Photoaging tends to be delayed in SOC, but uneven skin tone and post‐inflammatory pigment alterations (PA), including hyperpigmentation and hypopigmentation, are major concerns [10, 11, 12, 13]. Aethetic interventions in SOC require greater attention to risk of pigments alterations and propensity for hypertrophic/keloidal scarring [2]. Periprocedural skincare has been shown to improve outcomes and patient satisfaction with aesthetic procedures [12, 14, 15].

The current algorithm aims to provide clinicians with periprocedural adjunctive skincare recommendations for SOC patients receiving skin rejuvenation treatments with nonenergy‐based treatments (e.g., chemical peels, injectables) or nonablative energy devices to optimize outcomes, prevent sequelae, reduce recovery time, and improve comfort.

## 2 Methods

The algorithm used a modified Delphi approach, which aims to obtain consensus among experts through multiple rounds of iterative processes. Through this, an algorithm is developed with input from the expert panel and current literature review.

### 2.1 Literature Review

Prior to the panel meeting, a structured literature review was conducted by AA and Hinke Andriessen (HA) on December 20–22, 2023, selecting best‐practice approaches for periprocedural skincare for nonenergy device, injectable, and nonablative energy‐based aesthetic facial procedures in patients with SOC. Inclusion criteria were English language clinical studies on humans, guidelines, algorithms, and reviews with current best‐practice literature on peri‐procedure measures and skincare in SOC* individuals treated with injectables** or nonablative energy devices*** published from 2010 to January 2024. Excluded were articles that did not deal with pre‐/post procedural skincare for individuals with SOC, treated with injectables nonablative energy devices, and published in a language other than English.

Search terms used for injectables, nonenergy devices, and nonablative devices were divided into three groups.

Group 1: SOC* patients treated with injectables**/chemical peels**/fillers** AND hyperpigmentation OR post‐inflammatory hypopigmentation AND.

Group 2: *Energy***/laser*** treatment AND wound healing OR hyperpigmentation OR hypopigmentation OR pigmented scars OR melasma.

Group 3: *SOC ** *** AND sunscreen OR skincare OR *combined with skincare OR hypochlorous acid OR topical hydroquinone OR topical tranexamic acid OR topical kojic acid OR niacinamide OR combinations.

Titles and abstracts were reviewed and then articles. Searches on PubMed and Google Scholar (secondary source) conducted for group 1 and group 2 yielded 178 papers on nonenergy and injectable treatments [62] and 85 on nonablative energy treatments. After excluding 31 papers (duplicates, not reporting on skincare), 147 remained that mainly discussed reduction of adverse events after procedures.

**FIGURE 1:**
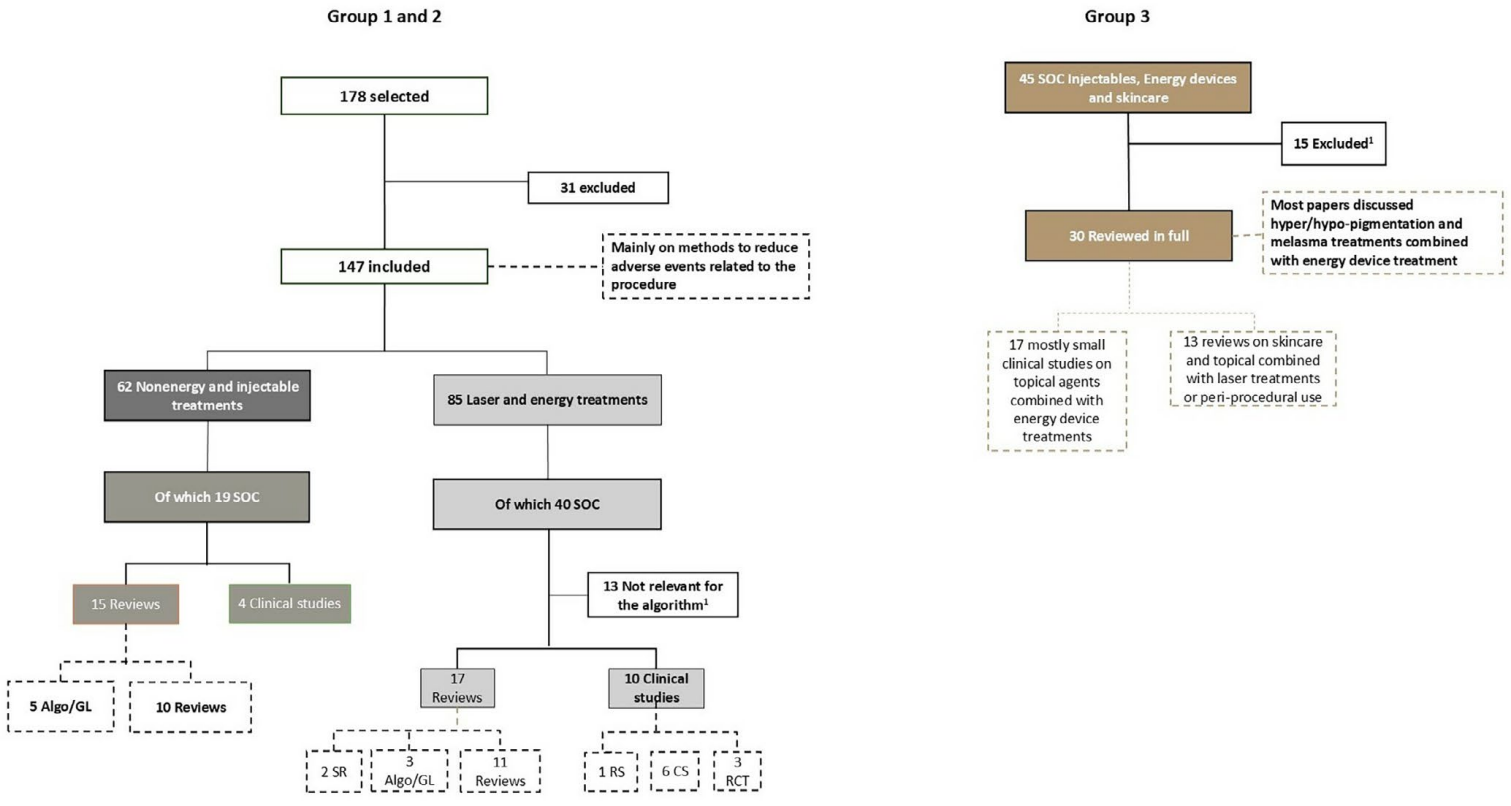
Structured literature search results. Algo, algorithm; CS, cross‐sectional studies; GL, guidelines; MA, meta‐analysis; RCT, randomized controlled trials; RS, retrospective studies; SR, systematic reviews. ^1^Excluded: Not including injectables, nonablative laser, periprocedural skincare, SOC patients. Due to a lack of clinical studies on periprocedural skincare in SOC patients, no grading was done.

Searches on PubMed and Google Scholar (secondary source) for group 3 yielded 45 papers on SOC patients receiving facial treatment with nonenergy and injectable treatments, nonablative energy devices, and integrated skincare. After excluding 15 papers (duplicates, not reporting on skincare or SOC), 30 remained that mainly discussed dyschromia and melasma treatments combined with energy device treatment. The papers included 17 small clinical studies on topical agents combined with energy device treatments and 13 reviews on skincare and topicals combined with laser treatments or periprocedural use (Figure 1). AA and AFA drafted an algorithm based on the results of the literature searches.

### 2.2 Role of the Panel

The panel comprised eight dermatologists from Brazil, Canada, South Africa, Mexico, and the USA. The international panel has extensive experience with medical aesthetic non‐energy and energy‐based procedures and has numerous publications on best practices in skin‐of‐color patients.

During the face‐to‐face meeting on March 8, 2024 in San Diego, after presentations of literature summaries, the panel worked in small groups to discuss and adapt the first draft of the algorithm. They then reconvened into a plenary group to customize the final algorithm and to reach a unanimous consensus (≥ 80% [7/8]) through blinded reiterations and votes. Preparation of the manuscript was done online.

## 3 Results

**FIGURE 2:**
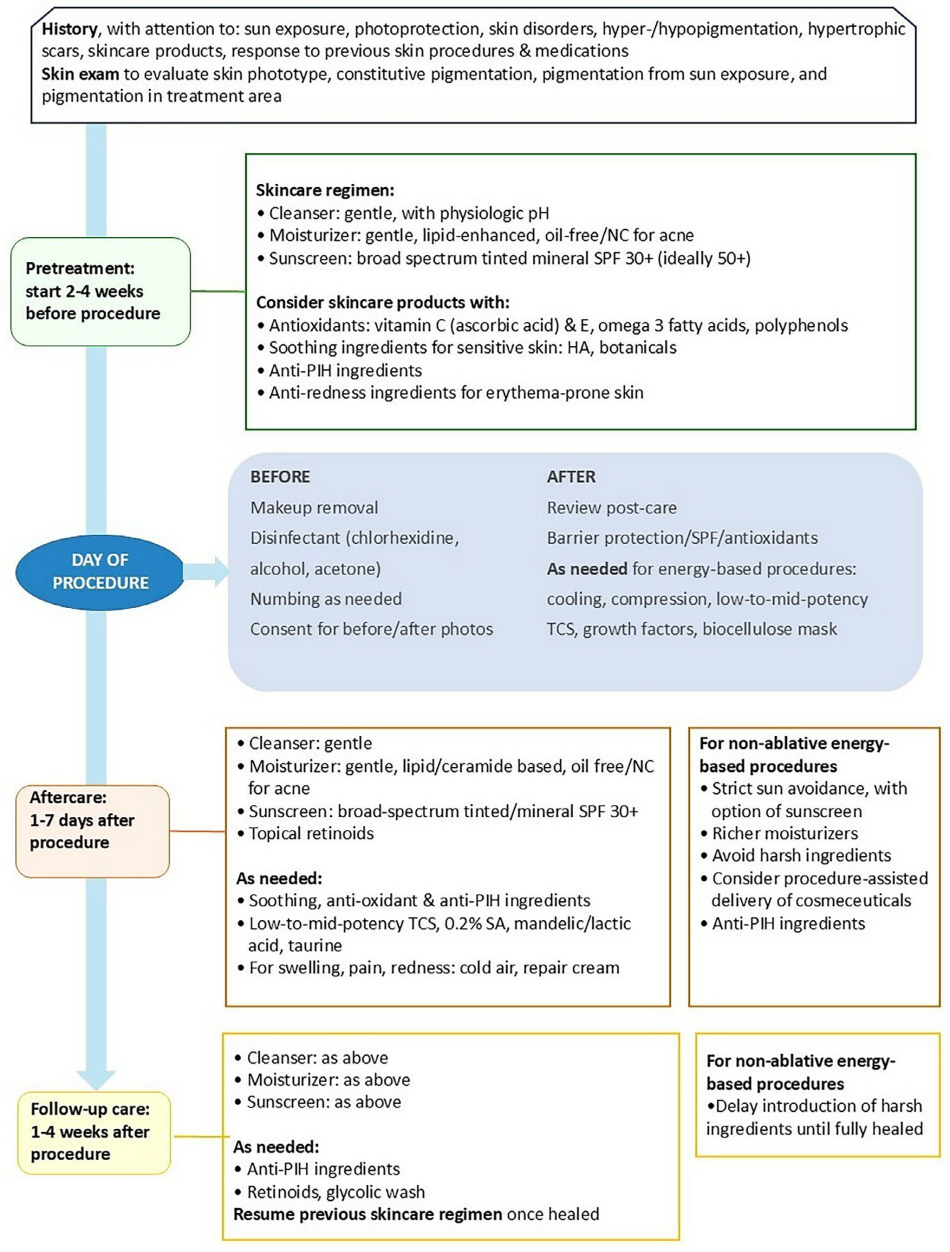
Periprocedural skincare for nonenergy and nonablative procedures in patients with skin of color. HA, hyaluronic acid; NC, noncomedogenic; PIH, post‐inflammatory hyperpigmentation; SA, salicylic acid; TCS, topical corticosteroids.

The algorithm has five sections, starting with a medical history and skin examination, followed by pretreatment measures beginning 2–4 weeks before the procedure, then measures on the day of the procedure, aftercare 1–7 days after the procedure, and follow‐up care 1–4 weeks after the procedure and ongoing (Figure 2).

### 3.1 History and Skin Examination

Patients' motivation and expectation for aesthic procedure should be appropriately explored prior to each procedure to ensure satisfactory outcomes [14, 15]. Baseline photographs are recommended by the panel, followed by a detailed discussion about side effects and potential sequellae [14, 15]. The patient's medical history is evaluated before an aesthetic procedure can be deemed safe. Medical conditions, medications, dietary supplements, and drug or topical product allergies are detailed. Patients with SOC may have distinct aesthetic concerns and have a higher risk of procedure‐associated sequelae, such as PA, hypertrophic scars, and keloids [5, 6, 7, 8, 9, 10, 11, 14, 15].

Patient response to previous facial antiaging treatments is also taken into consideration specifically their response to dermabrasion, chemical peels, and laser treatments [14, 15]. The panel agreed that skin examination includes scars, as patients with hypertrophic scars, keloids, or PA will need periprocedural cosmetic measures to reduce the risk of these complications [14, 15].

### 3.2 Pretreatment 2–4 Weeks Before the Procedure

Skin barrier‐related parameters evaluating transepidermal water loss (TEWL) and tape‐strippings to compare skin barrier strength showed low maturation and relatively weak skin barriers in East Asian and White women when compared to African‐Americans, who had low ceramide levels and high protein cohesion in the SC [16]. The differences in skin barrier properties may explain the increased skin reactivity observed in East Asians and the high prevalence of xerosis in black skin [16].

The panel recommended a gentle cleanser and moisturizer at all times [14, 15]. Proper skincare should be formulated with ingredients to improve stratum corneum (SC) hydration and restore skin barrier function [14, 15]. Lower pH (< 6.5) skincare promotes SC acidification, accelerates barrier recovery, and maintains barrier homeostasis [14, 15]. A cleanser should be fragrance and soap‐free and have a near‐physiological skin pH (< 6.5), and for those with acne, oil‐free and non‐comedogenic skincare should be used [14, 15]. Patients with SOC may require nuanced approaches to skincare due to potential racial/ethnic variations in physiologic and cultural factors related to skin hydration and its impact [16, 17, 18, 19]. Cultural norms related to cleansing and moisturization differ across diverse populations and need to be considered when offering skincare recommendations [10].

Depending on the patient's skin condition, topical products containing vitamin C, E, or polyphenols may be beneficial to reduce inflammation and PA [20]. Adjunctive or combined topicals may enhance aesthetic procedure outcomes (such as hyaluronic acid, botanicals, lipids or niacinamide) and may improve skin condition [16, 20, 21, 22].

Sun exposure is a significant contributor to PA. Despite the importance of sunscreen, there are few commercially available sunscreens designed for SOC, and finding a cosmetically elegant sunscreen for a SOC patient is challenging. A report indicated that people with SOC are less likely to use sunscreen and receive sunscreen recommendations from a dermatologist [17]. Broad‐spectrum, preferably tinted sunscreen, rich in antioxidants and free radical quenchers with a sun protection factor (SPF) of ≥ 50 is recommended for at least 4 weeks before the aesthetic procedure and is to be continued for ongoing protection [18]. Patients who demonstrate PA in response to procedures or trauma can be treated for two or more weeks before the procedure [5, 6, 10, 11, 12, 13, 14, 15, 17]. Topicals containing antioxidants, free radical quenchers, and tranexamic, kojic, azelaic, and glycolic acids to prevent PA may be beneficial [2, 10, 23, 24, 25, 26, 27]. A survey completed by 56 dermatologists and surgeons showed that topical hydroquinone for SOC was the preferred choice for PA prevention, together with diligent physical block sunscreen use (SPF of ≥ 50) and strict sun avoidance [25]. However, members of the panel did not routinely recommend hydroquinone for SOC due to a lack of evidence and geographic variations in regulatory factors related to hydroquinone (Table 1) [14, 15].

**TABLE 1:** Advisors' preferred pre‐/postprocedure skincare approaches.

Pre‐procedure	Post‐procedure
Gentle cleanser Moisturizer, ideally with niacinamide Sunscreen (usually physical)	Continue skincare and sunscreen
Antioxidants: Topical non‐esterified, acidic vitamins C, non‐esterified vit E, D‐alpha tocopherol, mineral selenium	Continue topicals at the discretion of the physician.
Consider risks/benefits and utility of topical retinoids, e.g., in some instances, pretreatment with topical retinoids may be recommended (for greater efficacy with resurfacing). In contrast, in others, they are discontinued (e.g., prior to a superficial chemical peel in SOC)	Patients return to retinoid within a week Glycolic acid for more sensitive patients Phyto gel to reduce inflammation For mechanical microneedling procedures, no post‐procedure products (except cleanser and moisturizer) for 24 h to avoid contact dermatitis
Natural lighteners, anti‐erythema ingredients, humectants Consider hydroquinone or non‐hydroquinone skin‐lightening agents for higher‐risk procedures (no consensus)	Consider hydroquinone or non‐hydroquinone skin‐lightening agents for higher‐risk procedures (no consensus)

In general, patients are recommended to withhold anti‐inflammatory drugs, topical retinoids, and tobacco for days or weeks prior to and after procedures to reduce bleeding and allow for wound healing [14, 15].

### 3.3 Day of Treatment

#### 3.3.1 Injectable and Nonenergy Treatments

There is no significant risk for PA or scar formation after dermal filler injections in SOC patients [11]. Mild and transitory adverse events may occur, such as edema, tenderness, erythema, bruising, and pain at the injection site [11]. When treating SFT IV, V, and VI, it is important to recognize differences in adverse events that may be experienced. Higher rates of PA have been reported in darker skin phototypes undergoing soft tissue fillers [11] and chemical peels [11].

#### 3.3.2 Nonablative Laser Treatment

The rising popularity of cutaneous lasers as an accepted antiaging therapy for all SPT has increased the demand [1, 5, 6, 9]. SOC is at a heighted risk for PA post‐procedure through three main mechanisms: (1) Increased incidental energy absorption by melanin in the skin, (2) melanocyte lability leading to postinflammatory hyperpigmentation or hypopigmentation, and (3) loss of pigment secondary to deleterious effects of the laser leading to decreased melanin production and melanocyte populations and subsequent hypopigmentation [5, 6, 9, 25, 26, 27, 28, 29, 30]. With optimal device selection, appropriate parameters, and pre‐/post‐treatment precautions, laser and light‐based treatments for hair removal, PA, skin resurfacing, and skin tightening can be used safely in patients with SOC [31]. Most data on lasers and light treatments in non‐white skin involve patients of East Asian ethnicity (e.g., Korean, Japanese, Chinese, Thai) [5, 6, 9, 15, 27, 28]. There is a lack of studies involving individuals of African ancestry or those with SPT V or VI. Careful selection of device and treatment parameters is required to minimize complications [27, 28, 29, 30].

### 3.4 Skin Preparation

The first step in skin preparation is universal removal of makeup and skin cleansing [14, 15]. Optimal cleaning agents include isopropyl alcohol, chlorhexidine, or hypochlorous acid (HOCl). Isopropyl alcohol is inexpensive and easy to obtain; however, it is flammable and can irritate the skin. Chlorhexidine is an effective cleanser but can be toxic to the eyes and ears [14, 15]. Stabilized HOCl is highly active against bacteria, viruses, and fungal organisms without the oto‐or ocular toxicity of chlorhexidine [32].

Pain management can be customized as needed at the discretion of the treating physician [14, 15].

### 3.5 Aftercare 1–7 Days After Treatment

There is an increased risk of PA with serial and fast injections and hypersensitivity to hyaluronic acid [11]. Avoid PA using low‐risk injection methods, such as slow injection times and threading versus serial puncture [14]. If PA occurs, the panel recommends a combination of topical lightening agents (tranexamic, kojic, azelaic, and glycolic acids or chemical peels, preferably salicylic acid or glycolic acid) and consistent mineral sunscreen use and sun avoidance [19, 23, 24]. If Hyaluronidase may be used to resolve PA related to a severe complication of a hyaluronic acid filler (e.g., vascular occlusion or inflammatory nodules) [14, 15]. Occasionally, PA may be the result of hemosiderin deposition in which Nd‐Yag lasers would need to be considered for clearance [14, 15].

Adverse reactions may be reduced by prompt epidermal cooling, providing pauses between laser passes to reduce bulk heating, as well as ice packs to the affected areas (Table 2) [25, 27, 29, 30, 31, 33, 34]. Reduction of inflammation using topical corticosteroids post‐treatment may be considered when significant post‐procedure erythema or edema is noted [33, 35].

**TABLE 2:** Considerations and approach to optimize energy treatment outcomes in SOC.

Consideration	Approach	References
Skin containing high amounts of melanin absorbs energy more efficiently than fair skin, but the absorption coefficient of melanin decreases markedly as wavelengths become longer.	Minimize risks with proper wavelength selection.	[20, 38, 39, 40]
Shorter wavelengths increase the risk of permanent PA and scarring due to melanin acting as a competing chromophore.		
Longer wavelengths penetrate more deeply into the dermis with less tissue damage and are not efficiently absorbed by melanin but may create skin inflammation, leading to PA.		
Patients with SOC require more conservative treatment.	Use lower fluences and longer pulse duration.	[20, 38, 39, 40]
A more conservative approach is needed for procedures such as laser hair removal and resurfacing.	Treatments require a greater number of sessions.	[20, 38, 39, 40]
Control and reduce skin heating and resultant skin injury.	Apply epidermal cooling with slower treatment speeds and pauses between passes.	[20, 38, 39, 40]

Abbreviations: PA, pigmentary alterations; SOC, skin of color.

### 3.6 Follow‐Up Care 1–4 Weeks After Treatment

Patients should be counseled to continue using gentle skincare and adequate sunscreen (SPF > 50) for a few weeks post‐procedure [14, 15, 17, 18]. Concurrent skincare regimens with neuromodulator injections have been shown to reduce improve outcomes in facial lines, pigmentation, and skin texture when compared to neuromodulator alone (Table 3) [16, 20, 21, 22, 23]. An expert consensus on periprocedural integrated skincare for noninvasive energy‐based device aesthetic procedures in clinical practice in China recommended skincare to improve skin condition and to reduce PA [36].

**TABLE 3:** Clinical evidence on Injectables and nonenergy treatment skincare approaches.

Type of treatment and skincare	References
Neuromodulator injections, HA filler, and HA skincare repeated combination treatment achieved greater change in global facial aesthetic appearance than monotherapy.	Cartier et al., 2020 [16, 23]
HA filler and neurotoxin injections combined with a topical skin treatment regimen leads to improvement in skin quality and aesthetic appearance.	Dayan et al. 2018 [20]
Neuromodulator injections, a hydroquinone skincare regime, and daily topical retinoids improved signs of photoaging.	Schlessinger et al. 2018 [21]
Combining neuromodulator injections for antiaging treatment with skincare containing retinol adenosine and HA optimized total treatment outcomes.	Ascher et al. [22]
Niacinamide inhibits melanosome transfer to keratinocytes and may be combined with TXA. Pre‐procedure and follow‐up skincare with niacinamide, KA, AzA, and TXA‐containing skin care may be recommended to improve outcomes.	Hollinger et al. 2018 [19]
A randomized, double‐blind, vehicle‐controlled study showed improvement in irregular facial hyperpigmentation [30]	Lee Do et al. 2014 [23]
KA is a radical oxygen scavenger and inhibits tyrosinase. A study compared a combination of topical KA and glycolic acid with topical hydroquinone 4% and found superior results for the KA and glycolic acid product.	Drealos et al. 2010 [24]

Abbreviations: AzA, azelaic acid; HA, hyaluronic acid; KA, kojic acid; TXA, tranexamic acid.

Few studies show whether PA can be minimized after facial energy‐based treatment. In a split‐face study of 40 Asian patients with SPT IV, short‐term use of post‐procedure topical corticosteroids reduced the risk of postinflammatory hyperpigmentation following fractional CO_2_ laser for acne scars [35]. Topical corticosteroids‐treated sites showed significantly reduced proinflammtory hyperpigmentation a few months later compared to the non‐treated sites [35].

Energy‐based device facial treatment may be combined with pre‐procedure topical antioxidants (15% vitamin C serum, 10% vitamin C serum, and botanical serum) to optimize treatment outcomes [31, 34, 36, 37, 38, 39, 40]. A split‐face study conducted in Brazil, the UK, and the US, treating the whole face with fractional laser comparing topical vitamin C, vitamin E, and ferulic acid serum post‐laser for 7 days compared to vehicle showed the regime promoted tissue healing and was well tolerated [38].

A South Korean prospective split‐face study (*N* = 25) on laser‐assisted (low‐fluence Q‐switched 1064‐nm Nd:YAG) treatment delivery for melasma used a topical facial serum containing 3% tranexamic acid, 1% kojic acid, and 5% niacinamide on one half of the face versus laser alone on the other half. After five sessions at 2‐week intervals, the topical treatment side showed more improvement than the side without skincare [39]. Another small South Korean study using a formulation of vitamins C, E, and ferulic acid as an adjunct to Q‐switched 1064‐nm Nd:YAG laser facial treatment showed that the topical antioxidants may improve laser treatment outcomes and is safe and well‐tolerated (Table 4) [40].

**TABLE 4:** Clinical evidence on laser pre‐/postprocedure skincare approaches in SOC.

Type of treatment and skincare	Region/Country	Result	Reference
1444‐nm nonablative fractional diode laser ex vivo uptake in human donor skin. Pre‐procedure topical antioxidants (15% vit C serum, 10% vit C serum, Botanical serum).	China	Enhanced vitamin C uptake was 10 and 21 times, and botanical serum was 6 times compared to controls.	Wang et al. 2022 [37]
Prospective, single‐arm split‐face, double‐blind, controlled pilot study (*N* = 15) with moderate (Glogau scale 3) photodamage. Whole‐face fractional ablative laser. Topical vitamin C, vitamin E, ferulic acid serum post‐laser treatment topical 15% vit. C, 1.0% Vit. E, and 0.5% ferulic acid serum for 7 days compared to vehicle.	Brazil, UK, US	The topical treatment regimen compared to the vehicle after fractional laser correlated with wound healing and tolerated well.	Waibel et al. 2015 [38]
Prospective split‐face study (*N* = 25) on laser‐assisted (low‐fluence Q‐switched 1064‐nm Nd:YAG) treatment delivery for melasma. Topical facial serum containing 3% TXA, 1% KA, and 5% niacinamide on one half of the face versus laser alone on the other half.	South Korea	Five sessions at 2‐week intervals. Topical facial serum is safe and effective when combined with laser to treat melasma.	Park et al. 2021 [39]
Single‐blinded, prospective, randomized split‐face study (*N* = 18, aged 26–53 years). Combination of vitamins C, E, and ferulic acid antioxidant formula as an adjunct to Q‐switched 1064‐nm Nd:YAG laser treatment.	South Korea	Adjuvant skincare treatments with antioxidants may improve laser treatment outcomes and are safe and well‐tolerated.	Kim et al. 2020 [40]

Abbreviations: HA, hyaluronic acid; KA, kojic acid; TXA, tranexamic acid.

### 3.7 Integrating Skincare Into Practice

Integrating periprocedural skincare for facial nonenergy and nonablative energy‐based procedures in patients with SOC is beneficial as it enhances treatment outcomes and patient experience and may reduce downtime. Choosing the correct skincare depends on the patient and treatment factors, and the product should be customized to the patient's cultural preferences. Educating clinicians and patients on suitable skincare and giving out samples for patients to test cosmetic acceptability and preference may enable an informed choice and avoid disappointment. Various skincare products have multiple and synergistic benefits that may suit patients' requirements.

### 3.8 Limitations

The algorithm recommendations presented were created from expert opinion and current literature. While alternatives exist for periprocedure skincare, the proposed algorithm provides a set of best practices developed by a panel of expert clinicians and supported by evidence in the literature.

## 4 Conclusions

SOC aesthetic procedures require specific skills in an experienced provider to provide high quality, aesthetic outcomes. The algorithm presented a stepwise process for optimal periprocedural skin care in patients with SOC who undergo facial aesthetic procedures with injections, nonenergy, and nonablative energy procedures. Periprocedural integrated skin care with gentle cleansers, moisturizers, photoprotection, antioxidants, botanical serum, and, where appropriate anti‐PA agents may help improve treatment outcomes in SOC patients.

## Author Contributions

All authors (A.F.A., A.A., R.A.B., V.B.C., L.R.G., R.G.B., L.N., and M.L.) contributed to developing the manuscript, reviewing this work, and agreeing with the content.

## Conflicts of Interest

The authors declare no conflicts of interest.

## Data Availability

Data sharing not applicable to this article as no datasets were generated or analysed during the current study.

## References

[1] American Society of Plastic Surgeons , “National Plastic Surgery Statistics Report,” https://www.plasticsurgery.org/documents/news/statistics/2022/plastic‐surgery‐statistics‐full‐report‐2022.pdf.

[2] A. F. Alexis , In Procedures in Cosmetic Dermatology: Cosmetic Procedures in Skin of Color, 1st ed. (New York: Elsevier, 2024).

[3] T. B. Fitzpatrick , “Soleil et Peau,” Journal of Medical Esthetic 2 (1975): 33–34.

[4] O. R. Ware , J. E. Dawson , M. M. Shinohara , and S. C. Taylor , “Racial Limitations of Fitzpatrick Skin Type,” Cutis 105, no. 2 (2020): 77–80. 32186531

[5] A. F. Alexis , “Lasers and Light‐Based Therapies in Ethnic Skin: Treatment Options and Recommendations for Fitzpatrick Skin Types V and VI,” British Journal of Dermatology 169, no. Suppl 3 (2013): 91–97, 10.1111/bjd.12526. 24098905

[6] S. B. Kaushik and A. F. Alexis , “Nonablative Fractional Laser Resurfacing in Skin of Color: Evidence‐Based Review,” Journal of Clinical and Aesthetic Dermatology 10, no. 6 (2017): 51–67. 28979657 PMC5605208

[7] W. Manuskiatti , D. Triwongwaranat , S. Varothai , S. Eimpunth , and R. Wanitphakdeedecha , “Efficacy and Safety of a Carbon‐Dioxide Ablative Fractional Resurfacing Device for Treatment of Atrophic Acne Scars in Asians,” Journal of the American Academy of Dermatology 63, no. 2 (2010): 274–283, 10.1016/j.jaad.2009.08.051. 20633798

[8] N. P. Chan , S. G. Ho , C. K. Yeung , S. Y. Shek , and H. H. Chan , “Fractional Ablative Carbon Dioxide Laser Resurfacing for Skin Rejuvenation and Acne Scars in Asians,” Lasers in Surgery and Medicine 42, no. 9 (2010): 615–623, 10.1002/lsm.20974. 20976801

[9] S. Eimpunth , R. Wanitphadeedecha , and W. Manuskiatti , “A Focused Review on Acne‐Induced and Aesthetic Procedure‐Related Postinflammatory Hyperpigmentation in Asians,” Journal of the European Academy of Dermatology and Venereology 27, no. Suppl 1 (2013): 7–18, 10.1111/jdv.12050. 23205540

[10] S. C. Taylor , “Meeting the Unique Dermatologic Needs of Black Patients,” JAMA Dermatology 155, no. 10 (2019): 1109–1110, 10.1001/jamadermatol.2019.1963. 31433461

[11] M. Hosseinipour , “Dermal Fillers Strategies and Adverse Events Specific to Skin of Color. Pearls From the Expert,” Journal of Drugs in Dermatology 12, no. 5 (2021): 353, http://nextstepsinderm.com/derm‐topics/dermal‐filler‐strategies‐and‐adverse‐events‐specific‐to‐skin‐of‐color.

[12] M. Gold , A. Andriessen , D. J. Goldberg , et al., “Algorithm for Nonenergy and Injectable Treatment Pre‐/Post‐Procedure Measures,” Journal of Drugs in Dermatology 20, no. 11 (2021): s3–s10, 10.36849/jdd.1121. 34784133

[13] A. F. Alexis and J. O. Obioha , “Ethnicity and Aging Skin,” Journal of Drugs in Dermatology 16, no. 6 (2017): s77–s80. 29028856

[14] E. Lain , A. Andriessen , V. B. Campos , et al., “A Practical Algorithm Integrating Skincare With Nonenergy and Injectable Dermatologic Procedures to Improve Patients Outcomes and Satisfaction,” Journal of Drugs in Dermatology 23, no. 4 (2024): 227–232, 10.36849/JDD.7918. 38564400

[15] E. Lain , A. F. Alexis , A. Andriessen , et al., “A Practical Algorithm Integrating Skincare to Improve Patients Outcomes and Satisfaction With Energy‐Based Dermatologic Procedures,” Journal of Drugs in Dermatology 23, no. 5 (2024): 353–359, 10.36849/JDD.8092. 38709701

[16] H. Cartier , P. Hedén , H. Delmar , et al., “Repeated Full Face Aesthetic Combination Treatment With AbobotulinumtexinA, Hyaluronic Acid Filler and Skin‐Boosting Hyaluronic Acid After Monotherapy With AbobotulinumtexinA, Hyaluronic Acid Filler,” Dermatologic Surgery 46, no. 4 (2020): 475–482, 10.1097/DSS.0000000000002165. 31592825 PMC7147415

[17] H. Song , A. Beckles , P. Salian , and M. L. Porter , “Sunscreen Recommendations for Patients With Skin of Color in the Popular Press and in the Dermatology Clinic,” International Journal of Women's Dermatology 7, no. 2 (2020): 165–170, 10.1016/j.ijwd.2020.10.008. PMC8072489 33937484

[18] A. B. Lyons , C. Trullas , I. Kohli , I. H. Hamzavi , and H. W. Lim , “Photoprotection Beyond Ultraviolet Radiation: A Review of Tinted Sunscreens,” Journal of the American Academy of Dermatology 84, no. 5 (2021): 1393–1397. 32335182 10.1016/j.jaad.2020.04.079

[19] J. C. Hollinger , K. Angra , and R. M. Halder , “Are Natural Ingredients Effective in the Management of Hyperpigmentation? A Systematic Review,” Journal of Clinical and Aesthetic Dermatology 11, no. 2 (2018): 28–37. PMC5843359 29552273

[20] S. H. Dayan , T.‐V. Ho , J. T. Bacos , N. D. Gandhi , A. Kalbag , and S. Gutierrez‐Borst , “A Randomized Study to Assess the Efficacy of Skin Rejuvenation Therapy in Combination With Neurotoxin and Full Facial Filler Treatments,” Journal of Drugs in Dermatology 17, no. 1 (2018): 48–54. 29320587

[21] J. Schlessinger , J. Kenkel , and P. Werschler , “Further Enhancement of Facial Appearance With a Hydroquinone Skin Care System Plus Tretinoin in Patients Previously Treated With Botulinum Toxin Type A,” Aesthetic Surgery Journal 31, no. 5 (2011): 529–539, 10.1177/1090820X11411579. 21719866

[22] B. Ascher , C. Fanchon , L. Kanoun‐Copy , A. Bouloc , and F. Benech , “A Skincare Containing Retinol Adenosine and Hyaluronic Acid Optimizes the Benefits From a Type A Botulinum Toxin Injection,” Journal of Cosmetic and Laser Therapy 14, no. 5 (2012): 234–238. 23016532 10.3109/14764172.2012.712700

[23] D. H. Lee , I. Y. Oh , K. T. Koo , et al., “Reduction of Facial Hyperpigmentation After Treatment With a Combination of Topical Niacinamide and Tranexamic Acid: A Randomized, Double‐Blind, Vehicle‐Controlled Trial,” Skin Research and Technology 20, no. 2 (2014): 208–212. 24033822 10.1111/srt.12107

[24] Z. D. Draelos , M. Yatskayer , P. Bhushan , S. Pillai , and C. Oresajo , “Evaluation of a Kojic Acid, Emblica Extract, and Glycolic Acid Formulation Compared With Hydroquinone 4% for Skin Lightening,” Cutis 86, no. 3 (2010): 153–158. 21049734

[25] M. H. Gold , A. Andriessen , J. L. Cohen , et al., “Pre‐/Postprocedure Measures for Laser/Energy Treatments: A Survey,” Journal of Cosmetic Dermatology 19, no. 2 (2020): 289–295, 10.1111/jocd.13259. 31840388

[26] R. L. Quiñonez , O. N. Agbai , C. M. Burgess , and S. C. Taylor , “An Update on Cosmetic Procedures in People of Color. Part 1: Scientific Background, Assessment, Preprocedure Preparation,” Journal of the American Academy of Dermatology 86, no. 4 (2022): 715–725, 10.1016/j.jaad.2021.07.081. 35189254

[27] M. H. Gold , A. Andriessen , J. L. Cohen , et al., “Algorithm for Pre‐/Post‐Procedure Measures for Facial Laser and Energy Device Treatment,” Journal of Drugs in Dermatology 20, no. 1 (2021): s3–s11.33852254

[28] C. Dierickx , “Using Normal and High Pulse Coverage With Picosecond Laser Treatment of Wrinkles and Acne Scarring: Long‐Term Clinical Observations,” Lasers in Surgery and Medicine 50, no. 1 (2018): 51–55, 10.1002/lsm.22763. 29140537 PMC5813159

[29] O. A. Ibrahimi , N. Saedi , and S. L. Kilmer , “Laser‐Based Treatment of the Aging Face for Skin Resurfacing: Ablative and Nonablative Lasers,” in Part 3. Aesthetic Surgical Procedures (Elsevier Inc, 2015), 549–560, 10.1016/B978-0-323-26027-5.00034-6.

[30] A. M. Tremaine and M. M. Avram , “FDA MAUDE Data on Complications With Lasers, Light Sources, and Energy Devices,” Lasers in Surgery and Medicine 47, no. 2 (2015): 133–140. 25655709 10.1002/lsm.22328

[31] T. S. Alster and E. L. Tanzi , “Laser Surgery in Dark Skin,” Skinmed 2, no. 2 (2003): 80–85, 10.1111/j.1540-9740.2003.01664.x. 14673304

[32] M. H. Gold , A. Andriessen , A. C. Bhatia , et al., “Topical Stabilized Acid: The Future Gold Standard for Wound Care and Scar Management in Dermatologic and Plastic Surgery Procedures,” Journal of Cosmetic Dermatology 19, no. 2 (2020): 270–277, 10.1111/jocd.13280. 31904191

[33] S. Shah and T. S. Alster , “Laser Treatment of Dark Skin: An Updated Review,” American Journal of Clinical Dermatology 11, no. 6 (2010): 389–397, 10.2165/11538940-000000000-00000. 20866114

[34] H. Woolery‐Lloyd , M. H. Viera , and W. Valins , “Laser Therapy in Black Skin,” Facial Plastic Surgery Clinics of North America 19, no. 2 (2011): 405–416, 10.1016/j.fsc.2011.05.007. 21763999

[35] N. Cheyasak , W. Manuskiatti , P. Maneeprasopchoke , and R. Wanitphakdeedecha , “Topical Corticosteroids Minimize the Risk of Postinflammatory Hyper‐Pigmentation After Ablative Fractional CO_2_ Laser Resurfacing in Asians,” Acta Dermato‐Venereologica 95, no. 2 (2015): 201–205. 24854088 10.2340/00015555-1899

[36] C. Zhang , W. Song , B. Yu , et al., “Expert Consensus on Perioperative Integrated Skincare for Noninvasive Energy‐Based Device Aesthetic Procedures in Clinical Practice in China,” Journal of the European Academy of Dermatology and Venereology 38, no. 2 (2024): 26–36, 10.1111/jdv.19857. 38419560

[37] J. V. Wang , N. Ugonabo , and R. G. Geronemus , “Fractional Diode Laser Pretreatment,” American Society for Dermatologic Surgery 48 (2022): 258–259, 10.1097/DSS.0000000000003496. 34889214

[38] J. S. Waibel , Q.‐S. Mi , D. Ozog , et al., “Laser‐Assisted Delivery of Vitamin C, Vitamin E, and Ferulic Acid Formula Serum Decreases Fractional Laser Postoperative Recovery by Increased Beta Fibroblast Growth Factor Expression,” Lasers in Surgery and Medicine 48, no. 3 (2016): 238–244, 10.1002/Ism.22448. 26612341

[39] S. J. Park , J. W. Park , S. J. Seo , and K. Y. Park , “Evaluating the Tolerance and Efficacy of Laser‐Assisted Delivery of Tranexamic Acid, Niacinamide, and Kojic Acid for Melasma: A Single‐Center, Prospective, Split‐Face Trial,” Dermatologic Therapy 35, no. 3 (2021): e15287, 10.1111/dth.15287. 34962047

[40] J. Kim , J. M. Kim , Y. I. Lee , A. Almurayshid , J. Y. Jung , and J. H. Lee , “Effect of a Topical Antioxidant Serum Containing Vitamin C, Vitamin E, and Ferulic Acid After Q‐Switched 1064‐Nm Nd:YAG Laser Treatment of Environment‐Induced Skin Pigmentation,” Journal of Cosmetic Dermatology 19, no. 10 (2020): 2576–2582, 10.1111/jocd.13323. 32052907

